# Melatonin Enhances the Tolerance and Recovery Mechanisms in *Brassica juncea* (L.) Czern. Under Saline Conditions

**DOI:** 10.3389/fpls.2021.593717

**Published:** 2021-04-01

**Authors:** Hee-Soon Park, Elham Ahmed Kazerooni, Sang-Mo Kang, Abdullah Mohammed Al-Sadi, In-Jung Lee

**Affiliations:** ^1^School of Applied Biosciences, Kyungpook National University, Daegu, South Korea; ^2^Department of Crop Sciences, College of Agricultural and Marine Sciences, Sultan Qaboos University, Muscat, Oman

**Keywords:** salinity, melatonin, antioxidant enzymes, photosynthesis, amino acids, phytohormones, green mustard

## Abstract

Melatonin has been recently known to stimulate plant growth and induce protective responses against different abiotic stresses. However, the mechanisms behind exogenous melatonin pretreatment and restoration of plant vigor from salinity stress remain poorly understood. The present study aimed to understand the effects of exogenous melatonin pretreatment on salinity-damaged green mustard (*Brassica juncea* L. Czern.) seedlings in terms of oxidative stress regulation and endogenous phytohormone production. Screening of several melatonin concentrations (0, 0.1, 1, 5, and 10 μM) on mustard growth showed that the 1 μM concentration revealed an ameliorative increase of plant height, leaf length, and leaf width. The second study aimed at determining how melatonin application can recover salinity-damaged plants and studying its effects on physiological and biochemical parameters. Under controlled environmental conditions, mustard seedlings were irrigated with distilled water or 150 mM of NaCl for 7 days. This was followed by 1 μM of melatonin application to determine its recovery impact on the damaged plants. Furthermore, several physiological and biochemical parameters were examined in stressed and unstressed seedlings with or without melatonin application. Our results showed that plant height, leaf length/width, and stem diameter were enhanced in 38-day-old salinity-stressed plants under melatonin treatment. Melatonin application obviously attenuated salinity-induced reduction in gas exchange parameters, relative water content, and amino acid and protein levels, as well as antioxidant enzymes, such as superoxide dismutase and catalase. H_2_O_2_ accumulation in salinity-damaged plants was reduced by melatonin treatment. A decline in abscisic acid content and an increase in salicylic acid content were observed in salinity-damaged seedlings supplemented with melatonin. Additionally, chlorophyll content decreased during the recovery period in salinity-damaged plants by melatonin treatment. This study highlighted, for the first time, the recovery impact of melatonin on salinity-damaged green mustard seedlings. It demonstrated that exogenous melatonin supplementation significantly improved the physiologic and biochemical parameters in salinity-damaged green mustard seedlings.

## Introduction

Environmental problems, such as drought, salinity, and the rising global temperature, cause considerable restraints in agricultural production and threaten food security worldwide (Tester and Langridge, 2010). Approximately 20% of farmlands are affected by salt globally, and each year, this number steadily increases because of natural causes or irrigation practices, as well as excessive fertilization and plowing, which result in a decreased or no plant yield (Deinlein et al., 2014; Ke et al., 2016). High salt concentrations generate ion toxicity (mainly sodium ions), osmotic stress, and oxidative damage, and suppress many biochemical and molecular processes (Chen et al., 2018). Genetic improvement is a useful strategy for crop enhancement (Ke et al., 2016; Zhu et al., 2016); however, due to the complex and controversial issues that are connected to transgenic (GM) crops (Raman, 2017), a substitute strategy for improving stress tolerance and prolonging leaf endurance and longevity can result in important agricultural applications.

Melatonin (*N*-acetyl-5-methoxytryptamine) is an amphiphilic low-molecular-weight hormone with an indolic structure (Arnao and Hernández-Ruiz, 2014; Campos et al., 2019). Melatonin was originally identified in bovine pineal glands in 1958 (Chowdhury et al., 2008). This biomolecule influences several biological functions, such as the sleep–wake cycle, bone metabolism, innate immune system, seasonal reproduction, and emotional behavior (Tan et al., 2010; Carrillo-Vico et al., 2013; Shi et al., 2015a). There has been great progress in understanding melatonin’s role in plants since its existence was initially reported in vascular plants in 1995 (Dubbels et al., 1995; Van Tassel et al., 1995). Several studies on melatonin have proposed its possible physiologic actions in plants, including growth-stimulating activity, seed germination, flowering, and rooting, thereby acting in the same manner as auxin and indole-3-acetic acid (Arnao and Hernández-Ruiz, 2014; Wei et al., 2015). Endogenously synthesized melatonin performs a migratory role in coping with biotic/abiotic stresses. It confers stress tolerance toward heavy metals, ultraviolet radiation, salt, drought, and temperature fluctuations (Reiter et al., 2015; Nawaz et al., 2016). Melatonin also promotes the activity of scavenging enzymes, which leads to reducing and eliminating internal and environmental oxidative damages (ROS) (Arnao and Hernández−Ruiz, 2015; Li et al., 2015). It has been reported that pretreatment with exogenous melatonin preserves plant integrity; plays a recovery role in plants under stressful conditions, such as drought, salinity, heat, chemicals, cold, and oxidation; and retards natural aging and senescence (Li et al., 2012; Shi et al., 2015b; Zhang et al., 2015).

Green mustard (*Brassica juncea* L. Czern.) is widely consumed all over the world, especially in Asia, as food and spice (Whitley, 2013). This species of the family Brassicaceae has been cultivated due to its nutritional and economic values (Ashraf and Iram, 2005). The leaves and seeds of this plant have been traditionally used because of its medicinal values mainly for internal and external diseases, such as rheumatism, skin disease, and inflammation (Lee et al., 2007). Furthermore, several studies have characterized the antioxidant, anti-cancer, anti-diabetic, and antimicrobial effects of green mustard (Sim et al., 2008; Jo et al., 2018). In case of salinity tolerance in the Brassicaceae family, *B. juncea* is relatively sensitive. Its growth and yield can be markedly reduced under stressful conditions, such as salinity, drought, and high temperature (Ashraf and Iram, 2005). Therefore, developing salt tolerance in this species would be economically valuable.

Salt stress reduction through exogenous melatonin pretreatment has already been extensively studied (Wang et al., 2016; Li et al., 2017; Ke et al., 2018); however, only a few have investigated the effects of melatonin on salt stress recovery. To fill this gap, we conducted this study to evaluate the effect of melatonin on the recovery of salt-damaged green mustard seedlings. Two specific objectives were set. First, we aimed to characterize the appropriate melatonin concentration that was efficient against salinity-damaged plants. Second, we intended to investigate how exogenous melatonin application affects the physiologic and biochemical characteristics of salinity-damaged plants by presenting growth changes, photosynthetic rate, plant hormone contents, amino acid contents, etc.

## Materials and Methods

### Selection of the Appropriate Melatonin Concentration

Green mustard seeds were provided by Danong Korea Ltd., Gyeonggi-do, South Korea. The seeds were surface-sterilized in 2.5% sodium hypochlorite and tested for viability, according to a method previously described by Ke et al. (2018). The germinated seeds were sown in plastic pot trays (28 cm × 54 cm) filled with horticultural soil (Shinsung Mineral Co., Ltd., South Korea). Then, two to three seeds were planted in each pot. The mustard seedlings were pre-cultivated under natural light in a greenhouse at Kyungpook National University, Daegu, South Korea (35.53′°N, 128.36′°E), with 46.7% relative humidity (RH) and 25°C/19°C (day/night) temperature, and the seedlings were watered daily. Furthermore, 3-week-old seedlings with uniform sizes were transferred to pots (10 cm × 10 cm) and subjected to different treatments. Pre-cultivated seedlings were kept for 2 days to allow them to acclimatize. Then, all mustard seedlings were randomly divided into two groups: (i) normal control, grown with only distilled water (100 mL/plant); and (ii) salinity treatment, irrigated with 150 mM NaCl (100 mL/plant). Each treatment contained a triplicate of 15 plants each. Each group was treated for 7 days. Afterward, the salinity-stressed seedlings were treated with 0, 0.1, 1, 5, and 10 μM melatonin (100 mL/plant, once a day) on their roots for 16 days. The melatonin (ChemScene LLC., Monmouth Junction, NJ, United States) solution (10 μM, stock) was prepared by dissolving the solute in distilled water (2.32 mg × 1,000 mL), followed by dilution with distilled water to prepare different concentrations. Plants of each concentration were measured for several plant growth characteristics. Plant growth parameters, such as plant height, leaf length, and leaf width were measured after day 4 (4DAT), day 8 (8DAT), day 12 (12DAT), and day 16 (16DAT) of treatment. Moreover, the 1 μM melatonin concentration was found to be the most optimal concentration for further studies.

### Effect of Exogenous Melatonin on Salinity-Damaged Green Mustard

#### Plant Material and Growth Conditions

The experiments were performed from December 2018 to February 2019 in a growth chamber at Kyungpook National University. The plants were grown under the aforementioned conditions. Moreover, green mustard seeds were planted in plastic pot trays containing horticultural soil (Shinsung Mineral Co., Ltd, South Korea). Seedlings of similar size were selected after 3 weeks and then transferred to pots (10 cm × 10 cm). During the experiment, the temperature was maintained at a constant 22°C/20°C (day/night) level, with an RH of 60% and a light intensity of approximately 200 μmol m^–2^s^–1^.

#### Salinity Stress Pretreatments

In this part of the study, 2 days after transplanting, 3-week-old seedlings were subjected to different treatments. They were divided into two groups: the normal control group, irrigated with distilled water (DW) (100 mL); and the salinity treatment group, irrigated with 150 mM NaCl (100 mL). Each group was treated for 7 days, and after which, the groups of unstressed and salinity-stressed seedlings were subdivided into two groups with an equal number of seedlings. This contributed to the formation of four experimental groups: (1) normal control, irrigated with DW alone (100 mL); (2) normal control, irrigated with solution containing 1 μM melatonin (100 mL); (3) salinity pretreated (150 mM NaCl), irrigated with DW (100 mL); and (4) salinity pretreated, (150 mM NaCl), irrigated with solution containing 1 μM melatonin (100 mL). Each treatment was performed in triplicate with 12 plants each. The mustard seedlings were treated for 8 days, and sampling was performed at days 4 and 8 in each treatment. After recording the chlorophyll content, the harvested samples (shoots and leaves) were either freshly used or rapidly deactivated in liquid nitrogen and stored at –80°C.

### Assessment of Plant Growth Characteristics and Chlorophyll Content

To investigate the effects of each treatment on green mustard seedlings, several plant growth parameters were measured. These parameters included plant height, stem diameter, and leaf length/width, which were recorded over a period of 8 days, specifically on day 0 (0DAT), day 4 (4DAT), and day 8 (8DAT). A digital Vernier caliper and a ruler were used to measure stem diameter and plant height, respectively. For estimating the leaf area (length and width), a fully expanded leaf (beneath the growing point) from each plant was selected and measured using a ruler. Chlorophyll content in leaves was examined using a portable CCM-300 Chlorophyll Content Meter (ADC BioScientific Ltd., Herts, England). Each treatment had three replicates.

### Analysis of Photosynthetic Gas Exchange Parameters

The photosynthetic rate, stomatal conductance, and transpiration rate were determined using an LCpro T portable photosynthetic assay system (ADC Bioscientific Ltd., Herts, England). The plants were constantly exposed to photosynthetically active radiation (PAR) 1,500 μ mol m^–2^s^–1^. The topmost fully expanded leaf from each group was selected at the vegetative stage and measured on day 0 (0DAT), day 4 (4DAT), and day 8 (8DAT). The photosynthetic parameters were taken within the chamber between 9:00 and 11:00 AM, and each treatment had three replicates.

### Determination of Leaf RWC

Relative water content (RWC) was measured in the stressed and unstressed plants following the method previously described by Ahmad et al. (2019). This measurement was conducted for each treatment on day 4 (4DAT) and day 8 (8DAT). The fresh weight (FW) of the fourth leaf (counting from the bottom) of mustard seedlings was immediately recorded after harvesting (between 5:00 and 6:00 PM). Then, the leaf segments were made to float in DW in a closed container at 25°C for 15 h in the dark, and its saturated weight (SW) was determined. After that, the leaf samples were kept in the oven at 70°C for 48 h to obtain the dry weight (DW). Finally, the relative water concentration was estimated according to the formula RWC (%) = (FW-DW)/(SW-DW) × 100. Each treatment had three replicates.

### Quantitation of ABA Content

Abscisic acid (ABA) content was analyzed according to a previously described method (Qi et al., 1998; Kim et al., 2014). Freeze-dried sample of mustard leaves was ground using a mortar and pestle. The ground sample (approximately 0.1 g) was extracted with 10 mL of extraction solution. The filtered extract was concentrated, then dissolved in 5 mL of sodium hydroxide (1N NaOH), and washed three times with dichloromethane (10 mL CH_2_Cl_2_) to eliminate the lipophilic materials. The pH of the aqueous phase was adjusted to 3.5 using hydrochloric acid (6N HCl). Then, ethyl acetate was added to it and mixed by vortexing. The supernatant, ethyl acetate extract, was evaporated to dryness and dissolved in phosphate buffer (PH 8.0) to remove phenolic compounds. PVPP (polyvinylpolypyrrolidone) was added to the extracted solution (phosphate buffer) and kept on a shaker for 40 min at 150 rpm. The pH of the phosphate buffer was brought to 2.5 and partitioned into ethyl acetate. The ethyl acetate extract was evaporated to dryness. The dried residue was dissolved in dichloromethane (CH_2_Cl_2_), followed by passing through a silica cartridge (Sep-Pak; Water Associates, Milford, MA, United States) pre-washed with dichloromethane and MeOH diethylether (C_5_H_14_O_2_). Finally, the extract was dried with nitrogen gas (N_2_), and diazomethane (CH_2_N_2_) was added to it for methylation. ABA content was quantified through GC–MS (Agilent 6890N Gas Chromatograph, Santa Clara, CA, United States). A software (ThermoQuset, Manchester, United Kingdom) was used to observe the responses to ions [m/e of 162 and 190 for Me-ABA and 166 and 194 for Me-(^2^H_6_)-ABA].

### Quantitation of SA Content

Salicylic acid (SA) content was analyzed according to a previously described method (Enyedi et al., 1992; Seskar et al., 1998). Freeze-dried sample of mustard leaves was ground to a fine powder and approximately 0.1 g of it was extracted with methanol (90 and 100%) by centrifuging (12,000 rpm for 15 min at 4°C). The combined methanol extracts were vacuum-dried. The dried residue was dissolved in 5% trichloroacetic acid (TCA) and centrifuged at 10,000 rpm for 10 min. The supernatant was partitioned with ethyl acetate/cyclopentane/isopropanol (49.5:49.5:1, v/v). The top layer of the aqueous solution was dried and used for SA quantification through high-performance liquid chromatography.

### Measurement of Amino Acid Content

Quantification of amino acid content was carried out according to the described method of Waqas et al. (2015). Approximately 50 mg of the freeze-dried sample was hydrolyzed in the presence of hydrochloric acid (6N HCl, 1 mL) for 24 h at 110°C. Then, the extraction was concentrated and dried with vacuum at 80°C for 24 h. After that, the residue was diluted with deionized water (2 mL) and evaporated two times. Finally, the concentrated residue was dissolved with hydrochloric acid (0.02N HCl, 1 mL) and the mixture was passed through a 0.45-μm filter membrane. The solution was analyzed using a Hitachi L-8900 amino acid analyzer (Hitachi High-Technologies Corporation, Tokyo, Japan). Each treatment had three replicates.

### Measurement of Soluble Protein Content

Soluble protein content subjected to different treatments was quantified following the method previously described by Ashraf and Iram (2005). Fresh plant leaves (0.1 g) were ground to a fine powder using a mortar and pestle, and then mixed with 1 mL of phosphate buffer (50 mM, pH 7.0). The mixture was centrifuged at 10,000 rpm for 10 min at 4°C. Subsequently, the supernatant was collected and treated with the appropriate reagent, and the optical density was measured at 595 nm. Protein content was estimated in all the enzymatic preparations using the Bradford method (Bradford, 1976) with bovine serum albumin as standard.

### Determination of Enzymatic and Non-enzymatic Antioxidant Activity

Fresh leaf samples (0.1 g) were homogenized with 1 mL of ice-cold 50 mM phosphate buffer (pH 7.0, 1 mM EDTA, 1% PVP) and kept in an incubator at 4°C for 10 min. Subsequently, the mixture was centrifuged at 10,000 rpm for 10 min at 4°C. The supernatant was used for determining superoxide dismutase (SOD) and catalase (CAT) activity. SOD activity was measured using a SOD Assay Kit-WST (Dojindo Co., Ltd., Kumamoto, Japan). CAT activity was evaluated using the Amplex^TM^ Red Catalase Assay Kit (Thermo Fisher Scientific Korea Co., Ltd., Gangnam-gu, Seoul), according to the manufacturer’s instruction. DPPH radical scavenging activities, flavonoid and total phenolic content were analyzed by the method of Adhikari et al. (2018). The mixture activity and absorbance were measured using the Multiskan^TM^ GO UV/Vis microplate spectrophotometer (Thermo Fisher Scientific, Waltham, MA, United States) at a selected wavelength.

### Visualization of ROS by Staining

Accumulation of ROS components was determined based on the DAB (3,3-diaminobenzidine) staining method (Thordal-Christensen et al., 1997; Mishra et al., 2017). The third leaves were cut off on day 4 (4DAT) and day 8 (8DAT), and stained in the DAB solution (1 mg ml^–1^, pH 3.8). DAB staining and vacuum filtration were carried out for 12 h in darkness, followed by de-colorization of the stained leaves using absolute ethanol for 10 min in a boiling water bath. The leaves were then kept in 60% glycerol for 1-2 min. Three leaves were used for each treatment.

### Determination of ROS Content (H_2_O_2_)

H_2_O_2_ level subjected to several treatments was determined according to a previously described method (Jana and Choudhuri, 1982; Tsai et al., 2005). Third leaves of green mustard were collected on day 4 (4DAT) and day 8 (8DAT) and kept in a freezer at –80°C. The frozen leaves were ground with liquid nitrogen using a mortar and pestle. The ground sample (0.3 g) was homogenized with 3 mL of ice-cold phosphate buffer (50 mM, 1 mM EDTA, 1% PVP, pH 7.0) and centrifuged at 13,000 rpm for 20 min. The supernatant (2 mL) was mixed with 1 mL of 20% (v/v) H_2_SO_4_ containing 0.1% titanium chloride, and the mixture was centrifuged at 13,000 rpm for 20 min. The supernatant intensity was measured at 410 nm using the T60 UV-Vis spectrophotometer (PG Instruments Ltd., Wibtoft, United Kingdom). H_2_O_2_ level was determined using an extinction coefficient of 0.28 μmol^–1^cm^–1^. Three leaves were used for each treatment.

### Statistical Analysis

All experimental data were processed using ANOVA and Duncan’s multiple range test (*p* < 0.05) to examine the significant differences between the mean values. It was run with three biological replicates using Microsoft Excel 2017 and SAS statistical software (version 9.4, SAS Institute, Cary, NC, United States).

## Results

### Effects of Several Melatonin Concentrations on the Growth of Green Mustard Seedlings Under Salt Stress

**FIGURE 1:**
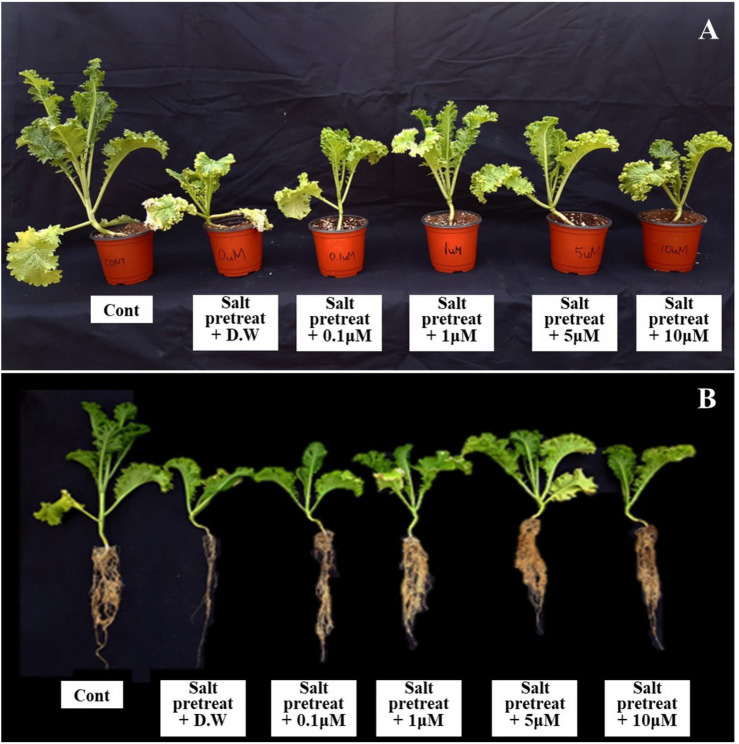
Effect of various melatonin concentrations on the growth of salt-stressed green mustard seedlings **(A,B)**. Treatment: control + water, 150 mM salt pre-treatment + water, 150 mM salt pre-treatment + 0.1 μM melatonin, 150 mM salt pre-treatment + 1 μM melatonin, 150 mM salt pre-treatment + 5 μM melatonin, 150 mM salt pre-treatment + 10 μM melatonin.

**FIGURE 2:**
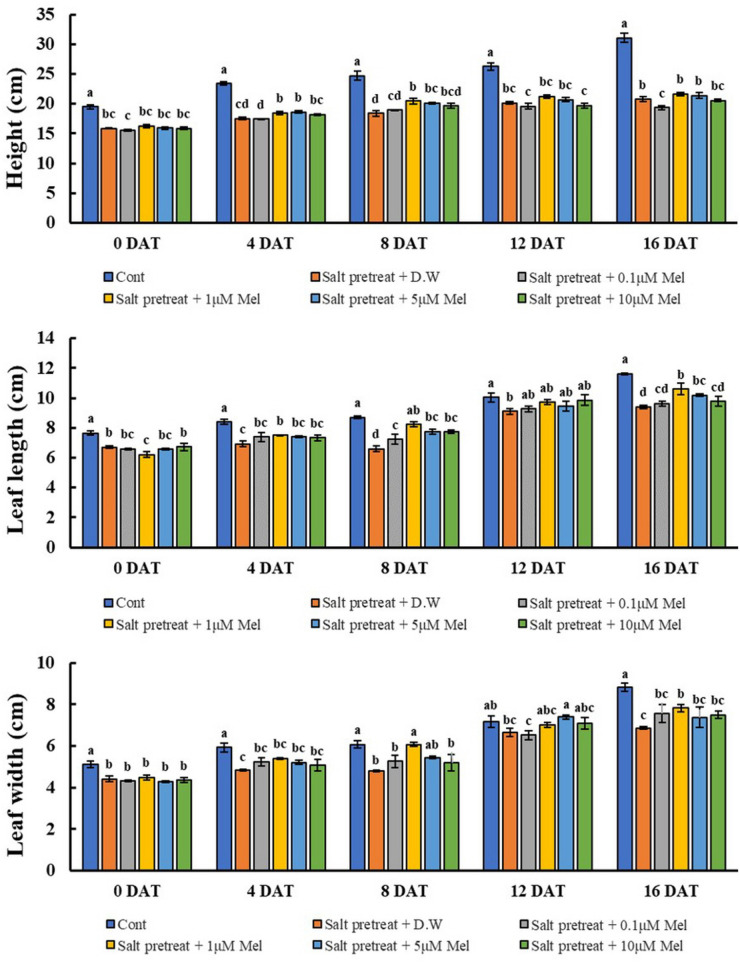
Effect of various melatonin concentrations on the growth of salt-stressed green mustard seedlings. The results were calculated from data for 0 (0DAT), 4 (4DAT), 8 (DAT), 12 (12DAT), and 16 (16DAT) days. Treatment: control + water, 150 mM salt pre-treatment + water, 150 mM salt pre-treatment + 0.1 μM melatonin, 150 mM salt pre-treatment + 1 μM melatonin, 150 mM salt pre-treatment + 5 μM melatonin, 150 mM salt pre-treatment + 10 μM melatonin. Values show the means ± SE (*n* = 3) and significant differences at *p* < 0.05 in accordance with Duncan’s multiple range tests.

The salinity treatment led to decreased plant height, leaf length, and leaf width, compared with the control. However, when salinity-stressed plants received several concentrations of melatonin (0, 0.1, 1, 5, and 10 μM), they generally showed alleviation of salt stress in comparison with salinity-stressed plants alone (Figure 1). Treatment with a lower concentration of melatonin (0.1 μM) showed no remarkable change, compared with salinity-stressed plants; or with a higher concentration of melatonin (10 μM), compared with salinity-stressed plants alone. However, salinity-stressed plants at a melatonin concentration of 1 μM revealed increased plant height (4.3%), leaf length (13%), and leaf width (11.2%) (*p* < 0.05) in contrast to salinity-stressed plants alone (Figure 2). These values showed that 1 μM of melatonin attenuated the suppressive effect of salinity stress on plant growth. Our findings indicate the necessity of choosing the appropriate melatonin concentration prior to application on crops.

### Recovery Effect of Exogenous Melatonin on Green Mustard Seedlings Under *Salinity Stress*

#### Effects of Melatonin on Plant Growth Characteristics

**FIGURE 3:**
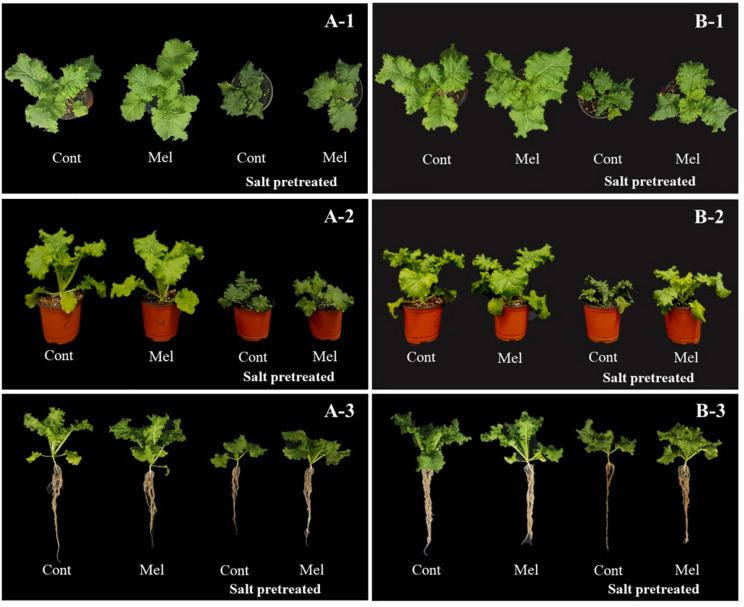
Effect of melatonin with/without salt stress on green mustard growth during four (4DAT; **A-1–3**) and eight (8DAT; **B-1–3**) days. Treatment: control + water, control + 1 μM melatonin, 150 mM salt pre-treatment + water, 150 mM salt pre-treatment + 1 μM melatonin.

Mustard seedlings were grown and supplemented with 1 μM melatonin concentration to examine the effects of exogenous melatonin on plant growth parameters. As shown in Table 1, no significant difference was observed between melatonin-treated and -untreated seedlings under normal conditions after 8 days. Salinity stress led to decreased plant height (34%), leaf length (32%), leaf width (34%), and stem diameter (20%) (*p* < 0.05). The growth of salinity-stressed plants with or without melatonin was inhibited, compared with unstressed plants. However, in melatonin-treated seedlings, plant height (10%), leaf length (16%), leaf width (20%), and stem diameter (13%) were higher than melatonin-untreated seedlings under salinity-stressed conditions. Our results showed that root application of 1 μM of melatonin improved the plant growth parameters under salinity stress (150 mM NaCl) conditions (Table 1 and Figure 3).

**TABLE 1:** The Effect of melatonin with/without salt stress on growth parameters in green mustard seedlings.

Treatment	Height (cm)	Leaf length (cm)	Leaf width (cm)	Stem diameter (cm)
**0 DAT**				
Cont	15.3 ± 0.4^a^	10.9 ± 0.2^a^	7.9 ± 0.1^a^	5.2 ± 0.1^a^
Mel	15.3 ± 0.1^a^	9.8 ± 0.5^b^	7.6 ± 0.1^a^	4.9 ± 0.0^b^
Salt pretreated Cont	10.1 ± 0.4^b^	7.1 ± 0.2^c^	5.2 ± 0.1^b^	4.1 ± 0.1^c^
Salt pretreated Mel	10.0 ± 0.1^b^	6.9 ± 0.1^c^	5.1 ± 0.1^b^	4.0 ± 0.0^c^
**4 DAT**				
Cont	19.1 ± 0.4^a^	12.8 ± 0.4^a^	8.4 ± 0.3^a^	6.1 ± 0.1^a^
Mel	19.4 ± 0.8^a^	13.0 ± 0.3^a^	8.7 ± 0.1^a^	6.2 ± 0.1^a^
Salt pretreated Cont	11.8 ± 0.2^c^	8.3 ± 0.2^c^	5.4 ± 0.1^c^	4.5 ± 0.1^c^
Salt pretreated Mel	13.0 ± 0.3^b^	9.4 ± 0.3^b^	6.3 ± 0.1^b^	4.9 ± 0.1^b^
**8 DAT**				
Cont	19.2 ± 0.4^b^	13.5 ± 0.5^a^	8.5 ± 0.1^a^	6.9 ± 0.2^a^
Mel	20.2 ± 0.5^a^	14.1 ± 0.2^a^	8.4 ± 0.1^a^	7.0 ± 0.1^a^
Salt pretreated Cont	13.6 ± 0.1^d^	9.4 ± 0.1^c^	6.1 ± 0.1^c^	5.2 ± 0.2^b^
Salt pretreated Mel	14.9 ± 0.3^c^	10.9 ± 0.3^b^	7.3 ± 0.2^b^	5.9 ± 0.2^b^

*The data were calculated from data for 0 (0DAT), 4 (4DAT), and 8 (8DAT) days. Treatment: control + water, control + 1 μM melatonin, 150 mM salt pre-treatment + water, 150 mM salt pre-treatment + 1 μM melatonin. Values indicate the means ± SE (n = 3). Letters represent significant differences at p < 0.05 in accordance with Duncan’s multiple range tests.*

#### Effects of Melatonin on Chlorophyll Content

**FIGURE 4:**
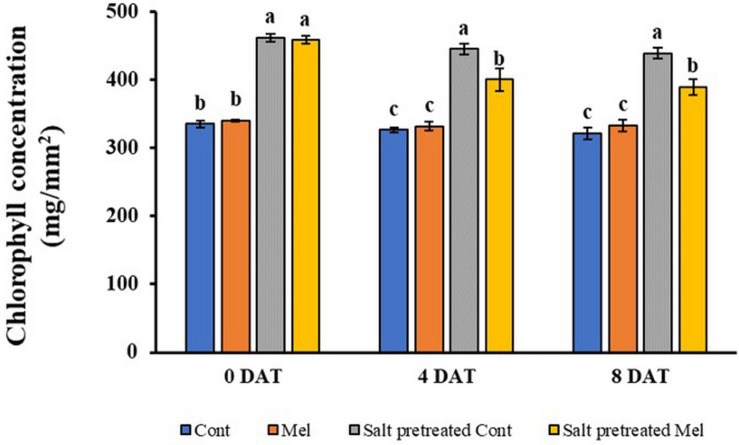
Effect of melatonin with/without salt stress on chlorophyll content in green mustard seedlings. The results were calculated from data for 4 (4DAT) and 8 (8DAT) days. Treatment: control + water, control + 1 μM melatonin, 150 mM salt pre-treatment + water, 150 mM salt pre-treatment + 1 μM melatonin. Values show the means ± SE (*n* = 3). Letters represent significant differences at *p* < 0.05 in accordance with Duncan’s multiple range tests.

As shown in Figure 4, no significant difference was observed between melatonin-treated and -untreated seedlings under normal conditions after 4 days (4DAT) and 8 days (8DAT). Salinity stress resulted in rapidly increasing chlorophyll content, compared with water-treated seedlings. Chlorophyll content was higher in salinity-stressed group plants by 36.5% (4DAT) and 36.4% (8DAT), compared with water-treated group (*p* < 0.05). Visual observations of salinity-stressed plants showed a deeper green leaf color. The root application of melatonin decreased the chlorophyll content by 11% in salinity-stressed green mustard seedlings. Our findings revealed that root application of 1 μM melatonin recovered the chlorophyll content under salinity stress (150 mM NaCl) conditions (Figure 4).

#### Effects of Melatonin on Gas Exchange Parameters

**FIGURE 5:**
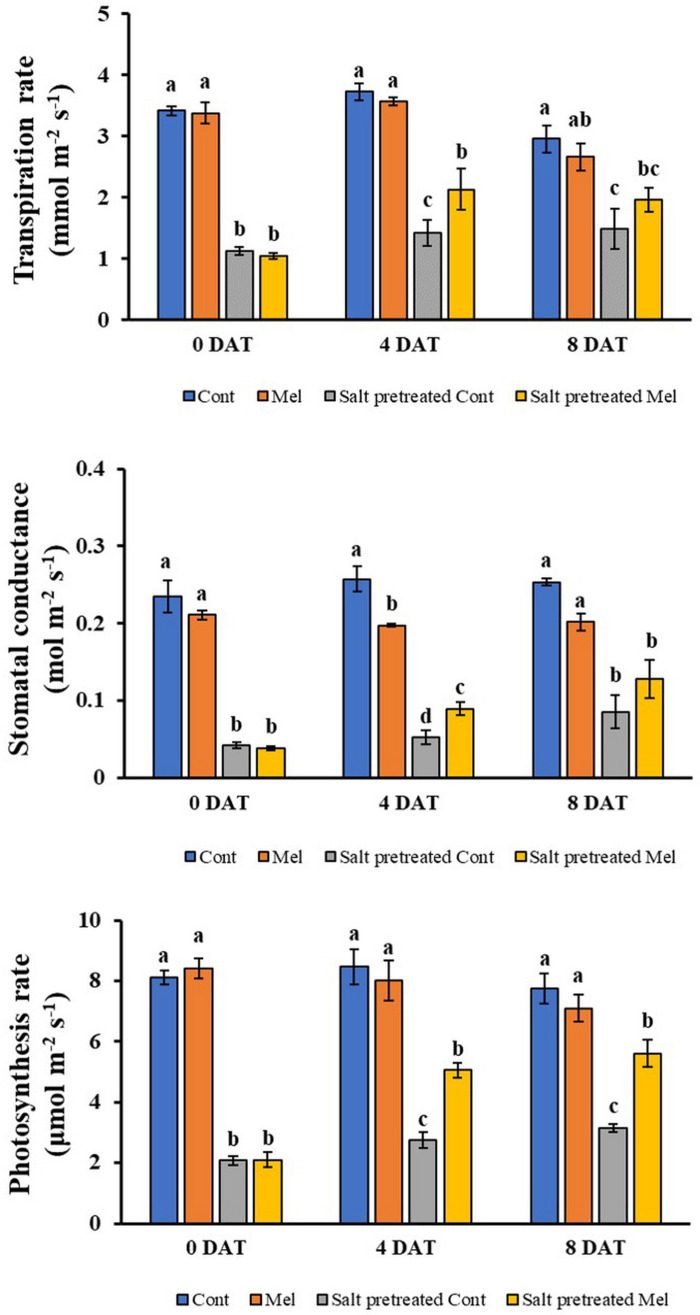
Effect of melatonin with/without salt stress on photosynthetic parameters in green mustard seedlings. The results were calculated from data for 4 (4DAT) and 8 (8DAT) days. Treatment: control + water, control + 1 μM melatonin, 150 mM salt pre-treatment + water, 150 mM salt pre-treatment + 1 μM melatonin. Values show the means ± SE (*n* = 3). Letters represent significant differences at *p* < 0.05 in accordance with Duncan’s multiple range tests.

A reduction in photosynthetic parameters was observed under salinity treatment (150 mM). Stomatal conductance, transpiration rate, and photosynthetic rate decreased significantly in salinity-stressed plants by 83, 67, and 74% (*p* < 0.05), respectively, compared with control plants (Figure 5). However, exogenous melatonin (1 μM) alleviated stress in plants grown under saline conditions. Notable enhancement in stomatal conductance (51%), photosynthetic rate (79%), and transpiration rate (32%) was detected when seedlings subjected to 150 mM salinity stress were treated with 1 μM melatonin over 8 days (*p* < 0.05) (Figure 5). Interestingly, the transpiration rate was higher on day 4 (9.18%) than on day 8 in response to melatonin–salt treatment. The decreased gas exchange parameters were alleviated through melatonin treatment in stressed plants over 8 days. Melatonin application particularly lowered stomatal conductance, photosynthetic rate, and transpiration rate in unstressed plants. This result indicated that root application of melatonin attenuated the salinity-induced reduction of photosynthetic parameters.

### Effects of Melatonin on Leaf RWC

**FIGURE 6:**
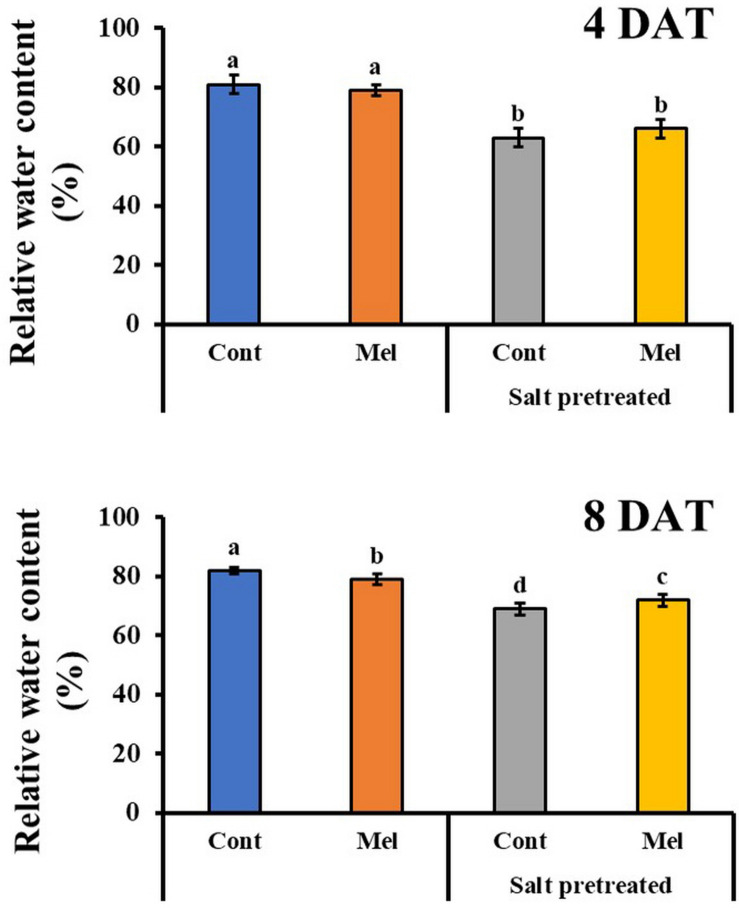
Effect of melatonin with/without salt stress on relative water content in green mustard seedlings. The results were calculated from data for 4 (4DAT) and 8 (8DAT) days. Treatment: control + water, control + 1 μM melatonin, 150 mM salt pre-treatment + water, 150 mM salt pre-treatment + 1 μM melatonin. Values show the means ± SE (*n* = 3). Letters represent significant differences at *p* < 0.05 in accordance with Duncan’s multiple range tests.

As shown in Figure 6, exogenous melatonin application caused a reduction in RWC of control plants on day 4 (4DAT) and day 8 (8DAT). A significant decrease (16%) in RWC of salinity-stressed seedlings was observed, compared with normal seedlings (Figure 6). On another note, there was a slight improvement (4.3%) in RWC when seedlings were exposed to 1 μM melatonin treatment and 150 mM salt stress over 8 days. These values showed that 1 μM melatonin relieved the suppressive effect of salt stress on the RWC content of mustard seedlings.

### Effects of Melatonin on ABA Content

**FIGURE 7:**
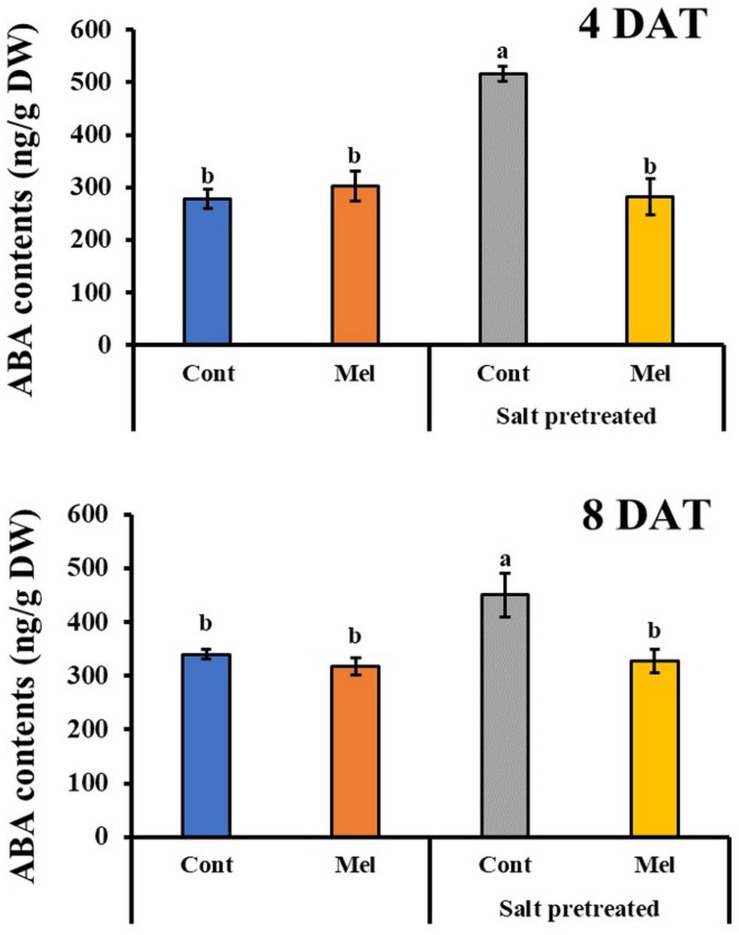
Effect of melatonin with/without salt stress on ABA content in green mustard seedlings. The results were calculated from data for 4 (4DAT) and 8 (8DAT) days. Treatment: control + water, control + 1 μM melatonin, 150 mM salt pre-treatment + water, 150 mM salt pre-treatment + 1 μM melatonin. Values show the means ± SE (*n* = 3). Letters represent significant differences at *p* < 0.05 in accordance with Duncan’s multiple range tests.

The endogenous ABA content was investigated over a period of 4 and 8 days to determine the influence of 1 μM melatonin treatment on the recovery of salinity-stressed (150 mM) mustard seedlings. ABA content significantly increased in the salinity-stressed seedlings, compared with normal seedlings. Whereas, by observing the ABA content at day 4 and day 8, we can see that it significantly decreased by 27% in salinity-stressed plants under 1 μM melatonin treatment (*p* < 0.05). On another note, ABA content was measured in plants under normal conditions with or without melatonin treatment. By day 4 (4DAT), ABA content of normal seedlings under 1 μM melatonin treatment slightly increased by 33%, compared with normal seedlings under 0 μM melatonin treatment (Figure 7). However, at the end of 8 days, ABA level in control plants receiving melatonin were lower than control plants without melatonin treatment. Our findings showed that 1 μM melatonin treatment resulted in a reduced ABA level under 150 mM salinity treatment.

#### Effects of Melatonin on SA Content

**FIGURE 8:**
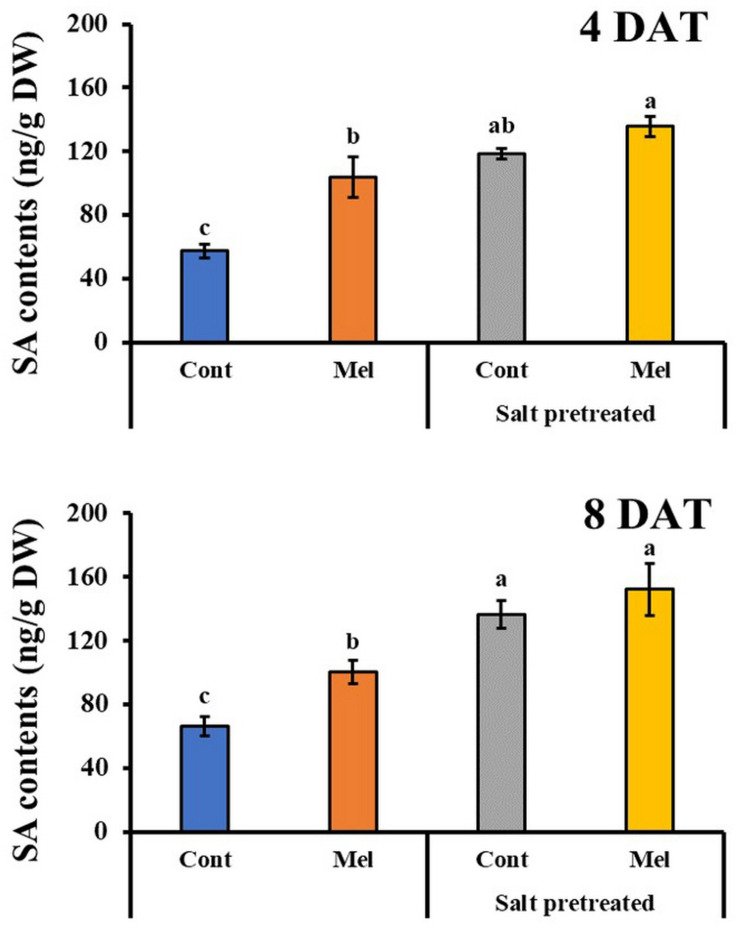
Effect of melatonin with/without salt stress on SA content in green mustard seedlings. The results were calculated from data for 4 (4DAT) and 8 (8DAT) days. Treatment: control + water, control + 1 μM melatonin, 150 mM salt pre-treatment + water, 150 mM salt pre-treatment + 1 μM melatonin. Values show the means ± SE (*n* = 3). Letters represent significant differences at *p* < 0.05 in accordance with Duncan’s multiple range tests.

Salicylic acid content in leaves was evaluated to examine whether melatonin treatment relieved the effects of salinity stress in mustard seedlings. In salinity-treated plants, SA content in mustard leaves increased by 106 and 106% on day 4 (4DAT) and day 8 (8DAT), respectively (Figure 8). As compared with salinity treatment alone, seedlings that were treated with 1 μM melatonin and 150 mM salt had elevated SA contents by 14% (4DAT) and 12% (8DAT) (*p* < 0.05). Similarly, normal seedlings that were treated with 1 μM melatonin for 8 days showed a remarkable 52% increase in SA content, compared with untreated plants (Figure 8). These values suggested that 1 μM melatonin application resulted in an increased SA content in seedlings that were pretreated with or without 150 mM salt.

#### Effects of Melatonin on Amino Acid Content

**FIGURE 9:**
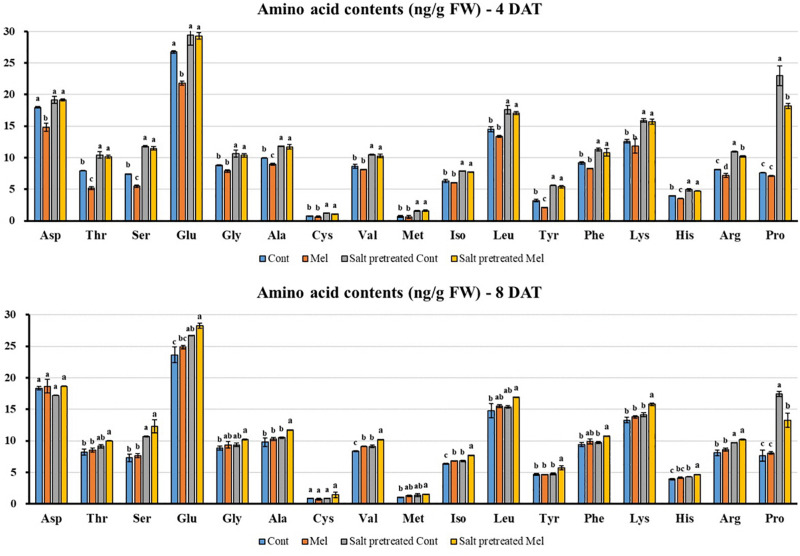
Effect of melatonin with/without salt stress on amino acid content in green mustard seedlings. The results were calculated from data for 4 (4DAT) and 8 (8DAT) days. Treatment: control + water, control + 1 μM melatonin, 150 mM salt pre-treatment + water, 150 mM salt pre-treatment + 1 μM melatonin. Values show the means ± SE (*n* = 3). Letters represent significant differences at *p* < 0.05 in accordance with Duncan’s multiple range tests.

Seventeen amino acids were detected with different concentrations in green mustard seedlings (Figure 9). Salinity stress increased amino acid content (except for Asp) in mustard seedlings, compared with seedlings under normal conditions over 8 days. Proline (Pro) dramatically increased by 128% in salinity-stressed plants. Additionally, amino acid constituents were recovered by root application of melatonin (except for Pro) in salinity-stressed plants on day 8 (8DAT). After 8 days, glutamic acid (Glu) had the highest value, whereas the lowest value was for cystine (Cys) (28.27–1.44 ng/g) in salinity pretreated plants subjected to melatonin treatment (Figure 9). The findings indicated that 1 μM melatonin application improved amino acid content under salinity-stressed (150 mM) and normal conditions.

#### Effects of Melatonin on Soluble Protein Content

**FIGURE 10:**
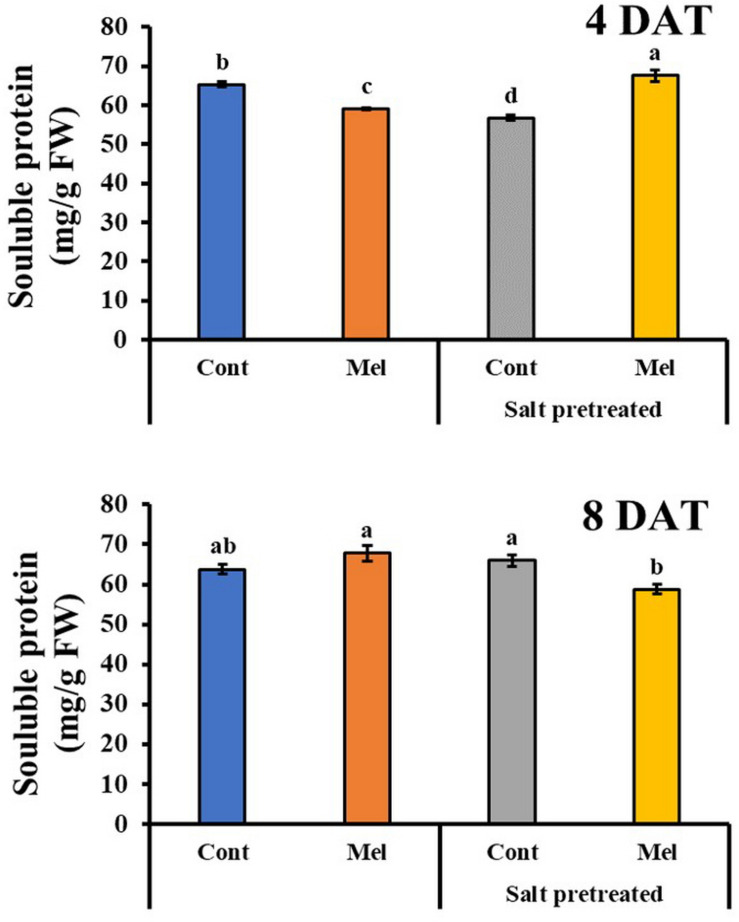
Effect of melatonin with/without salt stress on protein content in green mustard seedlings. The results were calculated from data for 4 (4DAT) and 8 (8DAT) days. Treatment: control + water, control + 1 μM melatonin, 150 mM salt pre-treatment + water, 150 mM salt pre-treatment + 1 μM melatonin. Values show the means ± SE (*n* = 3). Letters represent significant differences at *p* < 0.05 in accordance with Duncan’s multiple range tests.

Protein content decreased in salinity-damaged mustard over 4 days. By day 8, protein content slightly increased by 3.29% in salinity-damaged seedlings, compared with the control (*p* < 0.05) (Figure 10). During the fourth day, the application of 1 μM melatonin improved protein content by 19% in salinity-damaged plants, comparison with untreated salinity-damaged plants. However, by observing the results of protein content at day 8, we can see that the protein content decreased by 10.8% in salinity-damaged plants under 1 μM melatonin treatment (Figure 10). On another note, the protein content in control plants receiving melatonin were 6.3% higher than that in control plants without melatonin treatment. Our findings revealed that exogenous melatonin (1 μM) had a positive effect on protein accumulation under control and salinity stress conditions.

#### Effects of Melatonin on Antioxidant Activity

**FIGURE 11:**
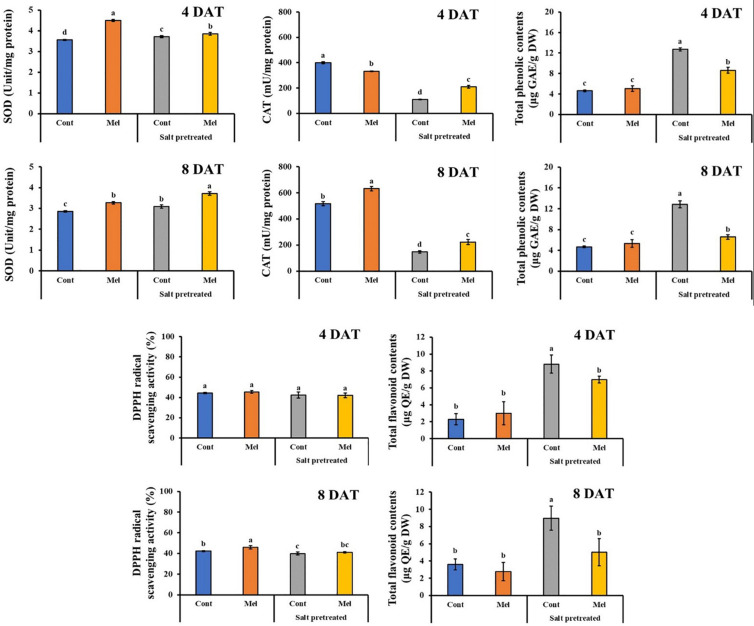
Effect of melatonin with/without salt stress on antioxidant content in green mustard seedlings. The results were calculated from data for 4 (4DAT) and 8 (8DAT) days. Treatment: control + water, control + 1 μM melatonin, 150 mM salt pre-treatment + water, 150 mM salt pre-treatment + 1 μM melatonin. Values show the means ± SE (*n* = 3). Letters represent significant differences at *p* < 0.05 in accordance with Duncan’s multiple range tests.

Compared with seedlings treated with water alone, melatonin application led to enhancement in antioxidant activity over 8 days (except. Flavonoid). SOD, CAT, DPPH, and total phenolic contents were raised by 14, 22, 8, and 12.66% in mustard seedlings under normal condition by the end of experiment (8DAT). However, flavonoid content in melatonin treated plants were 22.43% lower than untreated control plants by day 8 (*p* < 0.05). Additionally, salinity stress led to increase in SOD (8.4%), flavonoid (59.87%), and total phenolic (63.65%) contents, compared with untreated plants over 8 days. Whereas, Salinity stress (150 mM) led to a reduction in CAT (71.5%) and DPPH (5.54%) activity in mustard seedlings, comparison with control seedlings (*p* < 0.05). Compared with the seedlings treated with salinity alone, melatonin application contributed to additional increases in CAT and SOD activity by 51.7 and 19%, respectively over 8 days (Figure 11). On the other hand, total phenolic and flavonoid contents reduced in salinity-stressed plants receiving 1 μM melatonin treatment (Figure 11). There were no notable differences among melatonin-treated and -untreated plants exposed to salinity stress.

#### Effects of Melatonin on ROS by Use of Staining

**FIGURE 12:**
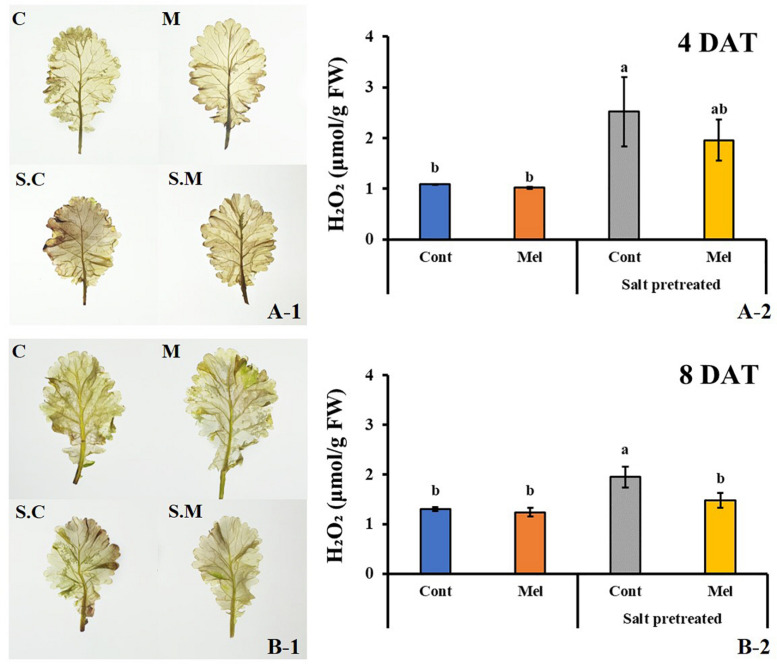
Effect of melatonin with/without salt stress on H_2_O_2_ content in green mustard seedlings. DAB staining was used to visualize the accumulation of H_2_O_2_ in leaves on day 4 (4DAT; **A-1**) and day 8 (8DAT; **B-1**) of treatment. The H_2_O_2_ contents were calculated from data for 4 (4DAT; **A-2**) and 8 (8DAT; **B-2**) days. Treatment: control + water, control + 1 μM melatonin, 150 mM salt pre-treatment + water, 150 mM salt pre-treatment + 1 μM melatonin. Values represent the means ± SE (*n* = 3). Letters represent significant differences at *p* < 0.05 in accordance with Duncan’s multiple range tests.

ROS accumulation was visualized for each treatment on day 4 (4DAT) and day 8 (8DAT) through DAB staining (Figures 12A-1,B-1). ROS accumulation under melatonin treatment decreased on day 4 and 8 in salinity-damaged mustard seedlings, compared with untreated damaged seedlings. The salinity-damaged plants were thoroughly stained by DAB, indicating high ROS accumulation. The intense brown color was higher in salinity-damaged plants, and this color intensity was clearly reduced in salinity-damaged plants supplemented with melatonin (Figures 12A-1,B-1). The DAB staining results unveiled high differences between treatments. The control plants treated with/without melatonin had less ROS accumulation than salinity-damaged plants. There were no significant differences among melatonin-treated and -untreated plants under normal conditions, and we observed a similar trend for leaf color in both groups (Figures 12A-1,B-1). Our findings revealed that 1 μM melatonin application led to decreased ROS accumulation in salinity-damaged plants.

#### Effect of Melatonin on ROS Content (H_2_O_2_)

The DAB staining results were confirmed by determining the H_2_O_2_ level in green mustard leaves subjected to different treatments. Our data agreed with the DAB staining results (Figures 12A-2,B-2). There was no noticeable difference between control plants with or without melatonin treatment. A slight decrease (4.9%) was observed in melatonin-treated plants under normal conditions; however, salinity stress remarkably enhanced H_2_O_2_ level by 49.7% in green mustard leaves, compared with the control group by the end of 8 days (*p* < 0.05). Salinity-induced H_2_O_2_ accumulation was decreased by melatonin application. Compared with the seedlings treated with salinity alone, melatonin application contributed to H_2_O_2_ level reduction by 24% over 8 days. Our results indicated that 1 μM melatonin application enhances the plants’ recovery from salinity damage through ROS detoxification in green mustard seedlings.

## Discussion

Salinity is one of the major abiotic stresses that affect plant growth and productivity (Jampeetong and Brix, 2009). Salt stress interferes with all the physiologic and metabolic processes in plants, including photosynthesis and protein synthesis (Passioura, 2010). Plants have established several strategies to deal with different environmental stresses. Recently, melatonin has emerged as a research focus due to its potential in plant physiology. Melatonin is also involved in several stress responses, such as being a plant growth modulator (Wei et al., 2015). Previous studies have indicated that exogenous melatonin can alleviate salt stress in plants (Mukherjee et al., 2014). In the present study, we examined the positive impacts of exogenously applied melatonin on the recovery of salinity-damaged green mustard seedlings.

The efficiency of melatonin has been proven to be concentration-dependent, and it may present a positive or negative impact on plants. For instance, drought-stressed coffee plants showed different responses to different doses of melatonin (Campos et al., 2019). Chen et al. (2009) reported that a low melatonin concentration accelerated root growth in *Brassica juncea*, whereas a high melatonin concentration suppressed it. Our findings showed that salinity-damaged seedlings supplemented with a melatonin concentration of 1 μM maintained higher plant height, leaf length/width, and stem diameter in contrast to salinity-stressed plants without melatonin treatment.

Changes in chlorophyll contents caused by salt stress depends on crop. Reduction in chlorophyll contents was observed in sunflower and rice under saline condition (Tatar et al., 2010). However, chlorophyll contents increased in wheat and several species of cotton that were exposed to salinity stress (Shah et al., 2017). Similarly, we observed that chlorophyll content increased rapidly in salinity-damaged mustard seedlings. This can be attributed to the adaptive mechanisms of plants to acclimatize to salinity stress and maintain its photosynthetic functions (Acosta-Motos et al., 2017). We found that melatonin treatment resulted in decreased chlorophyll production in salinity-damaged mustard seedlings during the recovery period. Considering that salt stress activates senescence in plants, the reduced chlorophyll content may be attributed to the protective effect of melatonin against chlorophyll degradation (Arnao and Hernández−Ruiz, 2009).

Photosynthesis is one of the most important physicochemical processes that are extremely salt-sensitive (Takahashi and Murata, 2008). Salt stress reduces stomatal opening, which leads to reduced CO_2_ diffusion to the mesophyll and impending changes in the photosynthetic rate. In the present study, we noticed that the photosynthetic parameters were remarkably lower in salinity-damaged plants, unquestionably because of stomatal limitations. However, exogenous melatonin application was found to improve stomatal conductance, and subsequently, the transpiration rate, photosynthetic rate, and leaf RWC were also increased in salinity-damaged seedlings during the recovery period (Hassanvand et al., 2019). Wang et al. (2016) reported that melatonin pretreatment of cucumber seedlings effectively enhanced their photosynthetic capacity under salinity stress. Similarly, melatonin pretreatment enhanced the salt tolerance of watermelon seedlings by decreasing stomatal limitation and increasing the photosynthetic rate (Li et al., 2017). Exogenous melatonin application generally contributes to a reduction in stomatal closure, and improvement in photosynthesis and water holding capacity under salinity stress.

Phytohormones not only influence the growth and development of plants, but they also effectively protect plants against biological and non-biological stresses (Hernández-Ruiz and Arnao, 2018). For instance, SA improved photosynthetic and growth parameters and antagonized oxidative damage in plants in response to abiotic stresses (Wani et al., 2017). Several studies indicated the impact of salt stress on phytohormone content, including SA and ABA (Zhang et al., 2015; Yang et al., 2017). The present study showed ABA and SA accumulation in the green mustard seedlings exposed to salinity stress. The rapid ABA and SA accumulation and the rapid reduction in stomatal size and conductance were found to be the bases for plant-induced tolerance to salt stress (Jakab et al., 2005). Furthermore, our findings showed that exogenous melatonin application can enhance the salt tolerance of green mustard by lowering its ABA content and elevating SA content, which is in accordance with recent reports (Figures 7, 8). Several studies demonstrate that exogenously applied melatonin downregulates the genes involved in ABA biosynthesis and subsequently upregulates the genes responsible for ABA catabolism/degradation, leading to a reduced ABA content and enhanced plant growth under stress conditions (Sharma and Zheng, 2019; Zhan et al., 2019). Environmental stress triggers the phenylalanine ammonia-lyase enzymatic pathway, which contributes to the induction and accumulation of endogenous SA (Wani et al., 2017; Pérez-Llorca et al., 2019). The increased melatonin levels in plants leads to enhanced SA levels, since melatonin performs an upstream of SA synthesis and induces SA biosynthetic genes (Lee et al., 2015).

Numerous studies have indicated the impact of environmental stresses on plant proteins (Su et al., 2014). To adjust the osmotic pressure, plants produce proteins and other metabolites that maintain cell balance (Kishor et al., 1995). In the present study, salinity stress caused hypoxia, and the soluble protein content decreased over 4 days. However, a slight increase in protein content was observed in salinity-damaged leaves by the end of experiment. This increase can be attributed to the plants’ stress response and adaptation to abiotic stresses (Chi et al., 2019). It has been observed that melatonin induced protein biosynthesis and prevents degradation; therefore, it sustains cell balance and physiologic activities (Chen et al., 2020). By the end of day 8 (8DAT), our findings showed that melatonin obviously raised the soluble protein content in green mustard seedlings under normal conditions. On day 4 (4DAT), we noticed an improvement in protein content in salinity-damaged plants with melatonin treatment, whereas, protein content clearly reduced on 8DAT in damaged seedlings treated with melatonin. This can be due to the stress-relieving effect of melatonin, which led to protein catabolism.

Plants usually produce high ROS under salt stress condition, which consequently causes oxidative damages, impaired membrane lipid functions, enzyme inactivation, and impeded metabolic activities (Huang et al., 2019). In the present study, we observed that H_2_O_2_ level were remarkably high in salinity-damaged plants, which agrees with previous reports. Exogenous melatonin application obviously mitigated the high H_2_O_2_ level in salinity-damaged plants by the end of 8 days (Figure 12B-2). This indicated that exogenously applied melatonin may efficiently protect cell membranes under salinity stress from oxidative damages. Owing to its solubility in either water or lipid, melatonin can easily cross the aqueous cytoplasm and lipid membranes, and prevent oxidative damages by deactivating and scavenging toxic substances (Venegas et al., 2012).

Plants have developed defensive mechanisms that consist of antioxidants with enzymatic or non-enzymatic activity to cope with oxidative damage and reduce excessive ROS accumulation. Several studies have demonstrated that melatonin upsurges the activity of enzymatic antioxidants under stressful conditions (Ahmad et al., 2019; Sharma and Zheng, 2019). Consistently, our results confirmed these previous works. Melatonin increased SOD, CAT, and DPPH activities in both unstressed and stressed seedlings. In the present study, the activity of CAT and DPPH reduced in the salinity-damaged plants, whereas flavonoid, SOD, and total phenolic content increased in the salinity-damaged plants, compared with the control plants. This shows the capability of antioxidants to rapidly react toward environmental stresses. Depending on the concentration of salt, SOD activity may increase, leading to scavenging excessive ROS and reducing oxidative damages (Song et al., 2006). Overall, our findings showed that the cross-talk among melatonin and antioxidants contribute to defer senescence in plants.

Amino acids directly or indirectly regulate plant responses to environmental stresses (Ashraf and Harris, 2004). In our experiment, salinity stress increased the amino acid contents (Glu, Ser, Thr, Arg, and Pro) in green mustard seedlings. This accumulation suggests that they have a role in osmotic adaptation apart from their roles in metabolism (Azevedo Neto et al., 2009). Several reviews have reported increased amino acid contents under salt stress (Rare, 1990; Mansour, 2000). Moreover, root application of melatonin rescued amino acid content in most cases (except for Pro) in salinity-damaged seedlings during the recovery period. The highest and lowest peaks belong to glutamic acid (Glu) and cysteine (Cys), respectively, in salinity-damaged plants with or without melatonin supplementation. Glu functions in nitrogen metabolism and is commonly used as a substrate for amino acid synthesis (Vance and Zaerr, 1990). Moreover, it plays a role in proline synthesis in response to abiotic stresses (Planchet and Limami, 2015). El-Shintinawy and El-Shourbagy (2001) found that the changes in Glu levels are correlated with Cys metabolism in plants under salt stress condition. These two amino acids act as a precursor of glutathione, a molecule that plays a crucial role in preventing ROS accumulation (Noctor and Foyer, 1998). Our results showed that root application of melatonin rescued Glu and Cys contents in salinity-damaged green mustard seedlings during the recovery period. This confirms the influence of melatonin on the metabolic pathways of these amino acids.

The Arg accumulation in green mustard subjected to salinity stress agrees previous works (Santa-Cruz et al., 1999). Rabe and Lovatt (1984) indicated that the increased Arg content in stressed plants can be due to *de novo* synthesis, because it is used for ammonia detoxification during reduced growth. The findings from this study showed that exogenous melatonin application led to an increased Arg content in salinity-damaged seedlings. Fan et al. (2015) showed the comprehensive influence of melatonin on several metabolic pathways (amino acids, organic acids, and carbohydrates) involved in plant resistance toward cold stress.

Leucine content decreased in salinity-damaged plants by day 8. El-Shintinawy and El-Shourbagy (2001) hypothesized that the reduction in leucine content may be due to the suppression of acetolactate synthase activity under salt stress. Additionally, previous reports demonstrated that changes in Leu content have also been correlated with protein catabolism/metabolism (Raggi, 1994); therefore, a decreased Leu content implies protein synthesis improvement. The findings from the present study showed that Leu accumulated in salinity-damaged green mustard under melatonin treatment (Figure 9). Shi et al. (2015a) reported Leu enhancement in melatonin-pretreated Bermuda grass under salt stress.

Several studies have been conducted on Asp accumulation in several plants species exposed to salt stress. They suggested that this accumulation can be used in salt-tolerant mechanisms and biosynthesis of the other amino acids (Azevedo Neto et al., 2009; Farhangi-Abriz and Ghassemi-Golezani, 2016). Our findings follow the same trend, as the Asp content increased in salinity-damaged seedlings for 4 days. After 8 days, the Asp content reduced in salinity-damaged seedlings, which may be due to the rapid Asp metabolism into the other products (Queiroz et al., 2012). Asp accumulation in salinity-damaged green mustard seedlings occurred after melatonin application. Melatonin-pretreated plant, *Cynodon dactylon*, enhanced Asp content under salt stress (Shi et al., 2015a). This demonstrates the effect of melatonin on amino acid metabolism.

A rapid increase was observed in the proline content in salinity-damaged green mustard, which is in agreement with the findings of previous studies on several plant species (Khattab, 2007; Bassuony et al., 2008). Accumulation of solutes called osmoprotectants, such as proline, may be a plant’s strategy to endure abiotic stresses (Dawood and El-Awadi, 2015; Liu et al., 2018). The increased proline content has been associated with enhanced plant performance under environmental stresses (Nanjo et al., 1999). Furthermore, proline may play a role as a reservoir of organic nitrogen that can be consumed during the recovery period to help plants withstand environmental stresses, such as salinity and drought (Rare, 1990; Sairam and Tyagi, 2004). In the present study, root application of melatonin led to a decreased proline content in salinity-damaged seedlings. Earlier studies indicated improvement in proline content in plants supplemented with a suitable concentration of melatonin under abiotic stresses (Liu et al., 2018; Ahmad et al., 2019). The observed decrease in proline content may be attributed to proline decomposition due to the stress-relieving effect of melatonin. Similarly, Zamani et al. (2019) reported that exogenous melatonin treatment reduced proline content in fenugreek (*Trigonella foenum-gracum* L.) under drought stress. As described previously, proline degradation is triggered in darkness and during stress relief, and is catalyzed by PDH (proline dehydrogenase) and P5CDH (Δ’-pyrroline-5-carboxylate dehydrogenase). Considering the alleviating impact of melatonin on oxidative damage caused by salt stress, proline degradation may be triggered during stress relief (Antoniou et al., 2017; Zamani et al., 2019). Additionally, proline degradation is needed to maintain growth and development under salt stress.

In a nutshell, our results confirmed that almost 5-week-old (38-day-old) salinity-damaged green mustard seedlings were recovered significantly by exogenous melatonin application. Our approach unraveled that exogenous melatonin can effectively improve performance and yield of mustard seedlings under either unstressed or stressed (salinity) conditions in an eco-friendly manner for a more sustainable agriculture. Future studies are needed to throw light on the genetic mechanisms and metabolic pathways during the recovery period in salinity-damaged green mustard plants under melatonin treatment.

## Data Availability Statement

The original contributions presented in the study are included in the article/supplementary material, further inquiries can be directed to the corresponding author/s.

## Author Contributions

H-SP and S-MK planned for research and analyzed the data. H-SP conducted the experiment. EK analyzed the data and wrote the manuscript. I-JL supervised the experiment and provided the resources. AA-S edited the manuscript. All the authors approved the manuscript.

## Conflict of Interest

The authors declare that the research was conducted in the absence of any commercial or financial relationships that could be construed as a potential conflict of interest.
